# Is *Mycoplasma pneumoniae* Adherence to Erythrocytes a Factor in Extrapulmonary Dissemination?

**DOI:** 10.1371/journal.ppat.1001219

**Published:** 2010-12-23

**Authors:** Harold Neimark, Matthew Gesner

**Affiliations:** 1 Department of Microbiology & Immunology, College of Medicine, State University of New York Health Science Center, Brooklyn, New York, United States of America; 2 Department of Pediatrics, College of Medicine, State University of New York Health Science Center, Brooklyn, New York, United States of America; The Fox Chase Cancer Center, United States of America

*Mycoplasma pneumoniae* is one of the most common causes of respiratory infections in children and adults worldwide [1], [2]. This bacterial pathogen is estimated to be responsible for at least one case of pneumonia per 1,000 persons and >100,000 adult hospitalizations per year in the United States alone [1]–[3]. The infection is mostly mild, but all age groups can experience more severe disease, and fatal cases occasionally occur.

Extrapulmonary manifestations are a notable aspect of *M. pneumoniae* infections and are seen in up to 25% of infected persons [1], [2], [4]. It has been pointed out that the high prevalence of *M. pneumoniae* infection in most populations predisposes to reporting concurrent but perhaps unrelated events as if they were part of the disease [2], and this may be so particularly for single case reports confirmed only by serologic response [1], [2]. Among reported extrapulmonary manifestations, joint, skin, hematologic, cardiovascular, nervous, and immune system disorders are solidly documented by culture, polymerase chain reaction (PCR), immunohistochemical analysis, and/or serologic analysis [1], [2], [4]–[7].

Characteristics of *M. pneumoniae* suggest the possibility that this mycoplasma could adhere to erythrocytes during extrapulmonary dissemination and such adherence could contribute to pathogenesis. First, *M. pneumoniae* has been cultured from extrapulmonary infection sites such as synovial fluid and pericardial fluid [1], [2], [4], so reaching such sites demonstrates that *M. pneumoniae* must be able to enter the blood stream. Second, it has long been known that *M. pneumoniae* adheres to human erythrocytes in vitro [8], [9], and electron microscopy shows *M. pneumoniae* does not merely adhere to erythrocytes but deforms them by producing depressions in the erythrocyte surface in which the mycoplasmas adhere closely [10]. Third, *M. pneumoniae* belongs to the same phylogenetic group that contains the hemotropic mycoplasmas; these uncultivated mycoplasmas parasitize erythrocytes of mammalian hosts and produce acute and chronic blood infections with hemolytic anemia and other illness [11]. Hemotropic mycoplasmas deform host erythrocytes and produce depressions in which they also adhere closely, and it is striking that the erythrocyte adherence of *M. pneumoniae* seen in vitro [10] appears to be identical to that observed in hemotropic mycoplasma erythrocyte infections [12]. Further, *M. pneumoniae* famously causes half or more of patients to produce erythrocyte cold agglutinins, and this character also is shared with hemotropic mycoplasmas [11]. We note that there is a report that a hemotropic mycoplasma can invade erythrocytes [13].

Some notion of the frequency of blood entry by *M. pneumoniae* may be provided by PCR studies that have demonstrated *M. pneumoniae* DNA in serum [14]–[16]. *M. pneumoniae* DNA has been detected in sera from pediatric patients both with pneumonia (1/25) and, significantly, without pneumonia (10/17), and in some cases *M. pneumoniae* DNA was detected for periods of more than 20 days, suggesting a bacteremia [14]. A real-time PCR study that utilized archival sera mainly from adults found *M. pneumoniae* DNA in 15 of 29 seropositive patient sera [16]. If these findings and other PCR reports detecting *M. pneumoniae* DNA in interior tissues often reflects the presence of organisms, as is thought by many investigators, then blood entry by *M. pneumoniae* might not be rare.

It may be necessary, nonetheless, to examine blood from a number of patients because of variables that could affect the presence of *M. pneumoniae* in a given sample. These include the frequency with which infection leads to blood entry, the infection stage(s) during which *M. pneumoniae* may enter the blood, the dwell period in blood, and the patient's immune status. The genotype of the infecting strain [17]–[20] also could be a factor in blood entry.

Rapid identification of *M. pneumoniae* infections by PCR [21]–[23] permits selection of appropriate cases for investigating the possibility that *M. pneumoniae* adheres to patient erythrocytes. The following information about hemotropic mycoplasma infections may be helpful in examining this possibility. Hemotropic mycoplasma infections have been detected mainly by visual search for erythrocyte-attached mycoplasmas in Wright-Giemsa blood smears, a relatively insensitive method, by animal inoculation, and by PCR (the current standard), but also of course by other molecular biological and instrument-based methods, including fluorescent-activated cell sorting. The percentage of infected erythrocytes in stained smears can vary from extremely high values (one or more mycoplasmas are seen attached to nearly every erythrocyte in most microscope fields) to very low values (searches of replicate smears from a PCR positive blood are negative), depending on, importantly, not only the stage of the infection but also the *Mycoplasma* species. Immuofluorescent or DNA staining substantially improves visual searching, and fluorescent staining allows more sensitive examination of archival Wright-Giemsa stained slides [24]. Specific staining also permits identification of mycoplasmas free in the plasma that by light microscopy might be mistaken for nonbacterial particles.

Obtaining proof that *M. pneumoniae* has the ability to adhere to patient erythrocytes would enlarge our understanding of *M. pneumoniae* pathogenicity and provide an intriguing new perspective on how this mycoplasma disseminates in the bloodstream to produce extrapulmonary disease.
